# D-lactate is a promising biomarker for the diagnosis of periprosthetic joint infection

**DOI:** 10.3389/fsurg.2022.1082591

**Published:** 2022-12-08

**Authors:** M. Fuchs, M. Faschingbauer, M. Riklin-Dold, P. Morovic, H. Reichel, A. Trampuz, S. Karbysheva

**Affiliations:** ^1^ RKU University Department of Orthopaedics, University of Ulm, Ulm, Germany; ^2^ Center for Musculoskeletal Surgery, Charité – Universitaetsmedizin Berlin, Berlin, Germany

**Keywords:** biomarker, periprosthetic joint infection, d-lactate, septic revision, diagnostic tool

## Abstract

**Introduction:**

Reliable biomarkers for the diagnosis of periprosthetic joint infection (PJI) are of paramount clinical value. To date, synovial fluid leukocyte count is the standard surrogate parameter indicating PJI. As D-lactate is almost solely produced by bacteria, it represents a promising molecule in the diagnostic workflow of PJI evaluation. Therefore, the purpose of this study was to assess the performance of synovial fluid D-lactate for diagnosing PJI of the hip and knee.

**Materials and Methods:**

These are preliminary results of a prospective multicenter study from one academic center. Seventy-two consecutive patients after total hip arthroplasty (THA) or total knee arthroplasty (TKA) were prospectively included. All patients received a joint aspiration in order to rule out or confirm PJI, which was diagnosed according to previously published institutional criteria. Synovial fluid D-lactate was determined spectrophotometrically at 450 nm. Receiver operating characteristic (ROC) analysis was performed to assess the diagnostic performance.

**Results:**

Eighteen patients (25%) were diagnosed with PJI and 54 patients (75%) were classified as aseptic. Synovial fluid D-lactate showed a sensitivity of 90.7% (95% CI: 79.7%–96.9%) and specificity of 83.3% (95% CI: 58.6%–96.4%) at a cut-off of 0.04 mmol/L. The median concentration of D-lactate was significantly higher in patients with PJI than in those with aseptic conditions (0.048 mmol/L, range, 0.026–0.076 mmol/L vs. 0.024 mmol/L, range, 0.003–0.058 mmol/L, *p* < 0.0001). The predominat microogranisms were staphylococci, followed by streptococci and gram-negative bacteria.

**Conclusion:**

D-lactate bears a strong potential to act as a valuable biomarker for diagnosing PJI of the hip and knee. In our study, a cutoff of 0.04 mmol/L showed a comparable sensitivity to synovial fluid leukocyte count. However, its specificity was higher compared to conventional diagnostic tools. The additional advantages of D-lactate testing are requirement of low synovial fluid volume, short turnaround time and low cost.

## Introduction

Worldwide, the numbers of total joint arthroplasty revision surgeries are constantly rising (1). One of the main indications for these procedures are periprosthetic joint infections (PJI), which occur in 0.3%–4% of all primary arthroplasties with even higher rates of up to 15% in revision surgeries (2–4). The diagnostic workflow of PJI evaluation includes various examinations, taking into account individual serum CRP levels, synovial leucocyte cell analyses as well as microbial and histopathological findings (5–7). While the existing PJI classifications lead to fair results, the correct diagnostic assessment of low grade infections in particular is challenging (8, 9). Consequently, current studies focus on the evaluation of new biomarkers in this theme (10–12). However, a single diagnostic tool with sufficient sensitivity and specificity is still missing. An explanation for this might be the fact, that the vast majority of recently investigated synovial biomarkers such as procalcitonin, alpha defensin, IL-1 and IL-6 are linked to the innate immunity (13–15). Therefore, these parameters are not specific for bacterial infections and can also be elevated in patients with systemic inflammatory diseases or within the early postoperative period (16–18). In contrast, the molecule D-lactate is the predominant form of lactate produced by different bacterial species. Due to the fact that it is almost solely produced by bacteria, D-lactate was shown to be a promising marker for the diagnosis of bacterial infections such as meningitis and septic arthritis (19).

Currently, a few studies evaluated the potential of D-lactate as a biomarker for PJI of the hip and knee. In 2019, Yermak et al. conducted a prospective observational study in which the authors reported about a similar performance of synovial fluid D-lactate concentration compared to synovial fluid leucocyte cell count for PJI assessment (20). With respect to the existing classification systems, Karbysheva et al. further reported about a sensitivity of D-lactate between 92%–94% and a specificity of 78%–89% in determining PJI (21). However, considering the defined quantitative thresholds, the three existing studies on this topic reveal significant variations. As such, the described synovial D-lactate cut-off differentiating between septic and aseptic conditions varies from 0.05 to 1.26 mmol/L (20–23). This heterogeneity according to the current state of relevant studies requires further scientific analyses. Therefore, the aim of this study was to evaluate the performance of D-lactate in synovial fluid as an independent diagnostic tool and define the optimal cut-off for diagnosing PJI.

## Material and methods

### Study design

These are preliminary results of a prospective multicenter study from one academic center. Between 1st of March 2020 and 1st of March 2021, consecutive patients aged 18 years or older who underwent a joint aspiration were considered for study inclusion. The following inclusion criteria were applied: (1) patients with a painful total hip (THA) or knee arthroplasty (TKA); (2) patients with progressive radiolucent lines after THA or TKA; (3) patients with scheduled THA or TKA revision surgery. The study was approved by the local ethics committee (registration number: FSta 40/20). Informed consent was obtained from all patients before participation. A standardized case-report form was used to collect patient history, demographic, clinical, radiological, microbiological, histopathological and laboratory data. All patients were evaluated by an interdisciplinary team consisting of orthopaedic surgeons and infectious diseases specialists. The study was performed in accordance with the Declaration of Helsinki. The D-lactate results were not communicated to the treating physician and thus did not influence individual infection management. A total of 121 participants were screened for study eligibility. Twenty-four synovial samples showed specimen clotting. Nineteen samples had an insufficient synovial fluid volume for laboratory analysis. Furthermore, 6 patients declined study participation. Thus, a total of 72 patients were included for further evaluation.

### Sample collection and preparation

Synovial fluid was aspirated under sterile conditions preoperatively in the outpatient department or intraoperatively during revision surgery *via* joint aspiration after skin incision and subcutaneous preparation before opening the joint capsule. Immediately after joint puncture, 1–3 ml of synovial fluid were inoculated into a pediatric blood culture bottles (BacTec PedsPlus/F, Beckton Dickinson and Co, USA) and 1 ml was introduced in a native vial for aerobic and anaerobic culture. An aliquot of 0.5–1 ml synovial fluid was collected in a native vial for D-lactate test, deproteinized and stored at −80°C until analysis. The remaining fluid was used for leucocyte count evaluation (1–2 ml). In cases of revision surgery, 3–5 periprosthetic tissue biopsies were collected intraoperatively from the implant-bone or cement-bone interface for microbiological and histopathological analysis. The explanted prostheses were collected in sterile containers and sent for sonication.

### Diagnosis of periprosthetic joint infection

PJI was defined according to previously published institutional criteria (21, 24–26). The different classification-based parameters include clinical features (visible purulence, presence of sinus tract), synovial fluid leukocytes (>2 × 10**^3^/**µl), granulocyte percentage (>70%), histopathology, and cultures of synovial fluid, periprosthetic tissue and sonication fluid. Cultures were considered positive if a high-virulent organism grew in ≥1 specimen of synovial fluid, periprosthetic tissue or sonication (*Staphylococccus aureus, Enterobacteriaceae, Streptococcus* spp., *Candida* spp.) or low-virulent organism grew in ≥2 specimen (*coagulase-negative staphylococci, enterococci, Cutibacterium* spp., *and other bacteria of the skin microbiome*). Sonication was considered positive if ≥1 CFU/ml of a high-virulent organism or >50 CFU/ml of a low- virulent organism grew in sonication fluid. The types of PJI were defined with regard to their temporal context in relation to the primary joint arthroplasty as previously described by Zimmerli et al. (5). Thus, the respective types of PJI were differentiated in early (those that developed less than 3 months after surgery), delayed (3 to 24 months after surgery) and late conditions (more than 24 months after surgery).

### Microbiological analysis of synovial fluid, sonication and periprosthetic tissue samples

One to three ml synovial fluid were inoculated in pediatric blood culture bottles and incubated at 36 ± 1°C for 14 days or until growth was detected. Additionally, synovial fluid samples of 0.1 ml aliquots were placed onto tryptic soy agar with 5% sheep blood for aerobic and anaerobic culture. The aerobic cultures were incubated at 37°C and inspected daily for 7 days, and the anaerobic ones were incubated for 14 days. The colonies of microorganism were identified by standard microbiological methods using automated system VITEK 2 (BioMérieux, Marcy L'Etoile, France). Tissue samples were cultured as described above. Sonication was performed according to a previously described protocol (27).

### Determination of synovial fluid D-lactate

D-lactate was determined in synovial fluid using D-Lactate Colorimetric Assay Kit (Abcam, Cambridge, UK). Laboratory analysis and sample preparation was performed according to the kit instruction. For the spectrophotometric assay, 40 µl of synovial fluid were used. A calibration curve with D-lactate standard solutions was calculated with each batch. After incubation at room temperature for 30 min, the optical density was measured at absorbance of 450 nm using a Microplate Absorbance Reader (DYNEX Technologies MRX, Chantilly VA, USA) and calculated to molar concentration using a calibration curve. The turnaround time amounts to 2 h.

### Statistical analysis

The significance level in all testing procedures was predetermined at *p* < 0.05. Quantitative data were presented as median and range or mean and standard deviation (SD), as appropriate. Statistical significance between the groups was assessed using the Mann–Whitney test. The sample size was calculated on the following assumptions: evaluation of the performance of D-lactate test in synovial fluid, assuming no difference margin of <10%, power 80% and α-risk 5%. Youden's J statistic was used for determining optimal D-lactate cut-off value on the receiver operating characteristic (ROC) curve by maximizing sensitivity and specificity. To compare the respective test performances, the area under the ROC curves were calculated for synovial fluid D-lactate, leukocyte count with granulocyte percentage, histopathology, culture and clinical features. All statistical analyses were performed using MedCalc 16.4.3 (MedCalc Software bvba, Ostend, Belgium). For graphical illustration, the software Prism (version 8.2; GraphPad, La Jolla, CA, USA) was used.

## Results

### Demographic data and PJI classification

In a total of 72 patients, 39 (54%) were male and 33 (46%) female. Among those, there were 55 patients (76%) with total hip arthroplasties and 17 patients (24%) with total knee arthroplasties. Eighteen patients (25%) were diagnosed with PJI. The majority of septic complications presented as delayed PJI (*n* = 9), followed by early (*n* = 5) and late (*n* = 4) infections. Fifty-four patients (75%) were classified as aseptic failure (AF), Table 1).

**Table 1:** Demographic data and infection characteristics of 72 patients.

Characteristics	All patients (*n* = 72)		
	PJI	AF	*p*-value
No. patients (%)	18 (25)	54 (75)	
Age, years, mean (range)	70 (54–86)	72 (40–90)	0.610
Male sex, No. (%)	11 (61)	28 (52)	0.497
Type of implant, No. (%)			
Knee	12 (67)	43 (80)	0.262
Hip	6 (33)	11 (20)	
Time from last surgery around the affected implant, months, mean (range)	30 (0.2–123)	89 (1–396)	0.008
Type of PJI, No. (%)			
Early (<3 months)	5 (28)		
Delayed (3–24 months)	9 (50)		
Late (>24 months)	4 (22)		
Patients with diabetes, No. (%)	3 (17)	10 (18)	0.859
Patients with underlying rheumatic joint diseases, No. (%)	1 (5)	4 (8)	0.789
Body mass index (kg/m^2^), mean (range)	28.9 (18.4–37.6)	29.3 (22.6–37.8)	0.816

AF, aseptic failure; PJI, periprosthetic joint infection.

### Performance of synovial fluid D-lactate

**Figure 1:**
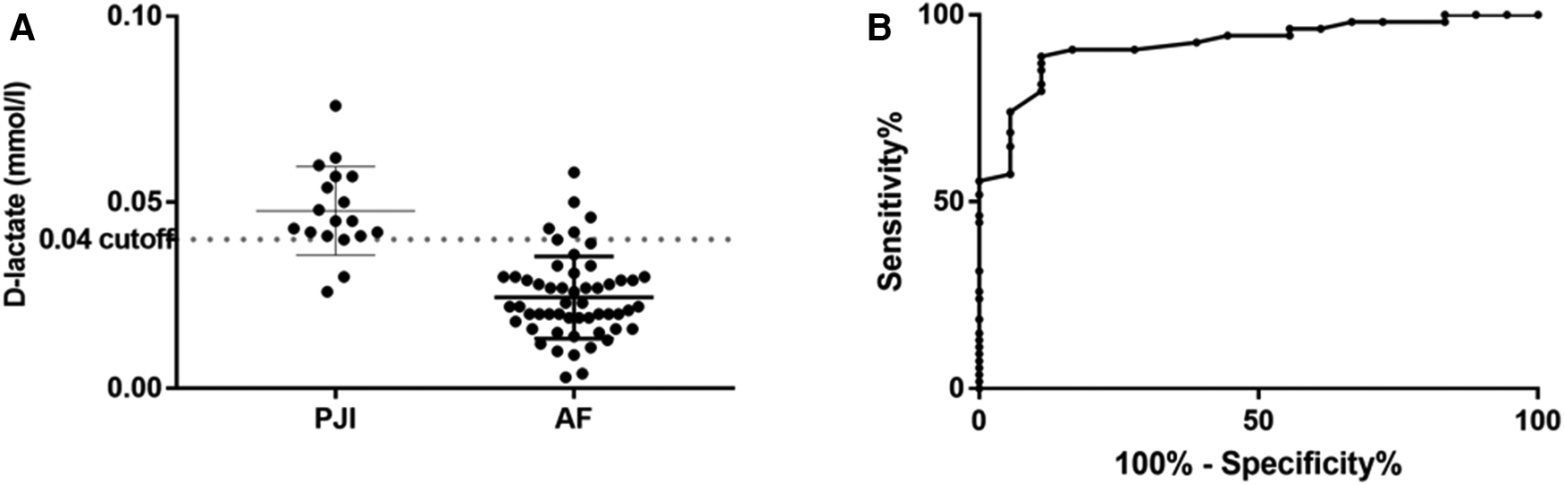
Distribution of D-lactate concentration in synovial fluid (**A**) with corresponding receiver operation characteristic (ROC) curve (**B**).

Synovial fluid D-lactate showed a sensitivity of 90.7% (95% CI: 79.7%–96.9%) and specificity of 83.3% (95% CI: 58.6%–96.4%) at a cut-off 0.04 mmol/L (Table 2). The median concentration of D-lactate was significantly higher in patients with PJI than in those with aseptic failure (0.048 mmol/L, range, 0.026–0.076 mmol/L vs. 0.024 mmol/L, range, 0.003–0.058 mmol/L, *p* < 0.0001) (Figure 1).

**Table 2:** Performance of different diagnostic criteria.

Criterion	Cut-off value	PJI	AF	AUC	Sensitivity, %	Specificity, %	PPV, %	NPV, %
					(95% CI)			
D-lactate, mmol/L	0.04	16/18	6/54	0.92 (0.86–0.99)	90.7 (79.7–96.9)	83.3 (58.6–96.4)	72.7 (55.2–85.2)	96.0 (86.6–98.9)
Purulence around the prosthesis or sinus tract communicating with the joint	–	5/18	0/54	0.64 (0.47–0.80)	27.8 (9.7–53.5)	100 (93.4–100)	100 (–)	80.6 (75.7–84.7)
Synovial fluid leukocytes, × 10**^3^/**µl and granulocytes, %	>2 >70	15/18	11/54	0.90 (0.79–1.00)	93.7 (69.8–99.8)	79.6 (66.4–89.4)	57.7 (44.2–70.1)	97.7 (86.5–99.6)
Histopathology of periprosthetic tissue samples	–	14/16	2/24	0.89 (0.78–1.01)	87.5 (61.6–98.4)	91.7 (73.0–98.9)	87.5 (64.7–96.4)	91.7 (74.9–97.5)
Positive culture samples								
Culture^a^	≥2	13/18	1/54	0.85 (0.72–0.98)	72.2 (46.5–90.3)	98.1 (90.1–99.9)	92.8 (64.6–98.3)	91.4 (83.4–95.7)

Note: If denominator is shown, the test was not performed in all patients.

PJI, periprosthetic joint infection; AF, aseptic failure; AUC, area under curve; PPV, positive predictive value; NPV, negative predictive value; CI, confidence interval; CFU, colony-forming unit.

^a^ Periprosthetic tissue, sonication and synovial fluid samples.

In 2 patients with PJI, the D-lactate test was false-negative. The diagnosis of PJI in these patients was based on clinical signs in combination with increased synovial fluid leukocyte count, positive histopathological and microbiological analyses (*E. coli* was detected in one patient). In patients with aseptic conditions, D-lactate was false-positive in 6 cases. Four of them had in addition increased synovial fluid leukocyte count which was not considered significant as these patients were diagnosed with periprosthetic fracture or luxation, polyethylene liner wear and one patient had a surgical intervention in the last 6 weeks. In the other two patients with false-positive D-lactate test, the diagnostic puncture was performed due to a painful prosthetic joint and progressive restriction of movement.

### Microbiological analysis

The isolated microorganisms mostly were presented by staphylococci, followed by streptococci and gram-negative bacteria (Table 3).

**Table 3:** Spectrum of pathogens.

Pathogen	PJI (*n* = 18)
Coagulase-negative staphylococci	4
*S. aureus*	1
*Streptococcus* spp.	4
*Enterococcus* spp.	1
Enterobacteriaceae	4
*Pseudomonas aeruginosa*	1
Other	–
Culture-negative	5
Polymicrobial infection	2

Table 3 illustrates the positive microbial results in patients with confirmed PJI. Among those, two individuals showed polymicrobial infections.

## Discussion

Defining synovial molecules that enable a reliable diagnostic workup of PJI considering biomarkers solely produced by bacteria is an innovative approach. Synovial fluid analysis determining leukocyte count and granulocyte percentage is the standard preoperative test with a sensitivity between 80%–86% and a specificity around 72%–93% (18, 28, 29). Although this analysis showed high sensitivity, it partially lacks specificity. Synovial fluid leukocyte count and granulocyte percentage may be elevated due to other inflammatory conditions in absence of infection such as periprosthetic fractures, underlying rheumatic diseases or within the early postoperative course. There are several studies evaluating the diagnostic impact of other promising molecules, such as alpha-defensin, leukocyte esterase, interleukin-6 and procalcitonin. However, the elevation of these parameters is not solely associated with bacterial infections. Consecuently, PJI diagnosis remains challenging, especially in patients with low-grade infections (30, 31). Therefore, a pathogen-specific biomarker would be of high clinical significance. The molecule D-lactate is almost solely produced by bacteria and showed a high sensitivity and specificity with regard to the current scientific evidence (20, 21). However, only few studies elucidate it's potential as a biomarker of bacterial infections with described cut-off values ranging from 0.05–1.3 mmol/L (20–23). Yermak et al. reported about a D-lactate cut-off of 1.26 mmol/L with sensitivity of 86.4% and a specificity of 80.8%. In their study, the authors evaluated 44 patients with PJI of the hip, knee or shoulder (20). Another work by Karbysheva et al. compared 2 different definition criteria (Musculoskeletal Infection Society, MSIS criteria and institutional criteria) and determined the optimal threshold of D-lactate for diagnosing PJI of the hip and knee (21). The authors defined a cut-off synovial fluid D-lactate concentration of 1.3 mmol/L, independent of the used definition criteria. The sensitivity of synovial fluid D-lactate was found to be 92.4%–94.3% with a specificity ranging from 78.4%–88.6% for the respective definition criteria. Li et al. conducted a meta-analysis to evaluate the diagnostic accuracy of D-lactate for PJI in which 5 studies were included (32). The pooled sensitivity and specificity of D-lactate for the diagnosis of PJI were 82% and 76%, respectively. However, this meta-analysis focuses on various anatomical locations as well as different PJI definition criteria. In the present study with regard to the diagnostic sensitivity and specificity of D-lactate, we observed similar findings. Synovial fluid D-lactate showed a sensitivity of 90.7% and specificity of 83.3%. Nevertheless, our cut-off of 0.04 mmol/L was substantially lower compared to the above mentioned publications. However, in all previously described studies, the measurement of D-lactate was performed spectrophotometrically by the use of different sample preparation procedures and test protocols. The applied wavelength varied from 340 to 570 nm depending on the study. These differences could partially explain the divergent cut-off values compared to the present results. ROC-curve analysis demonstrated that the AUC of D-lactate was higher or comparable to periprosthetic tissue culture, synovial leukocytes with granulocyte percentage and histopathology (*p* = 0.17, *p* = 0.38, and *p* = 0.34, respectively), but significantly higher than clinical features (*p* < 0.01, Table 2).

In our cohort, D-lactate was false-positive in 6 patients. The majority of these patients had increased synovial fluid leukocyte count due to different disorders other than infection (periprosthetic fracture, dislocation or surgical intervention in the last 6 weeks). These patients had no underlying disease such as severe uncontrolled diabetes or short-bowel syndrome which could lead to an increased D-lactate concentration in blood and body fluids (33). However, Yermak et al. (20) observed a positive correlation between elevated erythrocyte count and D-lactate in synovial fluid using spectrophotometric analysis. This could give an explanation for the false-positive D-lactate results due to a certain contamination of synovial fluid with blood components in patients with periprosthetic fracture, dislocation or within the early postoperative period. Additionally, polyethylene or metal particles in patients with component wear may influence the spectrophotometric analysis since the optical density of the sample is measured to calculate the concentration of analyte. Therefore, other more specific tests such as fluorimetric assay or liquid chromatography should be considered for D-lactate analysis in clinical samples (34, 35).

We are aware that our report has noteworthy limitations and leaves pending issues. First, the lack of patient follow-up examinations limits the value of this study. Second, the information about prior antibiotic use is not complete. Therefore, the effect of any antimicrobial therapy on D-lactate performance could not be reliably assessed. Finally, the small number of the patients included in the preliminary report leaves a number of questions open, e.g. the usefulness of the D-lactate test in patients with periprosthetic fracture, early postoperative period and liner wear. As our study focuses on preliminary results of a multicenter study, we hope to answer this question more specific in the future. In conclusion, our results reveal that D-lactate bears a strong potential to act as a valuable biomarker for the diagnosis of hip and knee PJI. In our study, a biomarker cutoff of 0.04 mmol/L showed comparable sensitivity to synovial fluid leukocyte count. However, as one may expect of a pathogen-specific biomarker, specificity was higher compared to previously published data of conventional diagnostic standards (36, 37). The main advantages of D-lactate testing are requirement of low synovial fluid volume, short turnaround time and low cost.

## Data Availability

The raw data supporting the conclusions of this article will be made available by the authors, without undue reservation.
