# Corrigendum: D-lactate is a promising biomarker for the diagnosis of periprosthetic joint infection

**DOI:** 10.3389/fsurg.2022.1122963

**Published:** 2023-01-12

**Authors:** Michael Fuchs, Martin Faschingbauer, Maximilian Riklin-Dold, Paula Morovic, Heiko Reichel, Andrej Trampuz, Svetlana Karbysheva

**Affiliations:** ^1^ Universitäts- und Rehabilitationskliniken Ulm, Ulm, Germany; ^2^ Charité Universitätsmedizin Berlin, Berlin, Baden-Wurttemberg, Germany

**Keywords:** biomarker, periprosthetic joint infection, D-lactate, septic revision, diagnostic tool

A Corrigendum on D-lactate is a promising biomarker for the diagnosis of periprosthetic joint infection By Fuchs M, Faschingbauer M, Riklin-Dold M, Morovic P, Reichel H, Trampuz A, Karbysheva S. (2022) Front. Surg. 9:1082591. doi: 10.3389/fsurg.2022.1082591

**Incorrect Funding**

In the published article, there was an error in the Funding statement. [This work was supported by an educational grant of the PRO-IMPLANT Foundation, Berlin, Germany (https://www.pro-implant.org), a non-profit organization supporting research, education, global networking, and care of patients with bone, joint or implant-associated infection.].

The correct Funding statement appears below.

**Funding**

[This work was supported by an educational grant of the PRO-IMPLANT Foundation, Berlin, Germany (https://www.pro-implant.org), a non-profit organization supporting research, education, global networking, and care of patients with bone, joint or implant-associated infection. We acknowledge financial support from the Open Access Publication Fund of Charité – Universitaetsmedizin Berlin and the German Research Foundation (DFG).]

The authors apologize for this error and state that this does not change the scientific conclusions of the article in any way. The original article has been updated.

